# Abnormal Brain Structural Covariance Networks of Cortical Thickness in Cocaine Use Disorder

**DOI:** 10.1111/adb.70176

**Published:** 2026-06-26

**Authors:** Linkai Chen, Youling Li, Jie Chen, Junshen Zhang, Xinyu Ma, Qiuling Cai, Guanghui Bai, Meimei Du

**Affiliations:** ^1^ Department of Radiology The Second Affiliated Hospital and Yuying Children's Hospital of Wenzhou Medical University Wenzhou China; ^2^ Wenzhou Key Laboratory of Structural and Functional Imaging Wenzhou China

**Keywords:** cocaine use disorder, cortical thickness, graph theory, rich club, structural covariance networks

## Abstract

Cocaine use disorder (CUD) is a serious global public health problem, characterized by compulsive drug seeking and impaired cognitive control. Previous studies using voxel‐based morphometry (VBM) or surface‐based morphometry (SBM) have identified regional alterations in grey matter volume and cortical thickness (CT); purely local morphological analyses are insufficient to capture coordinated patterns of interregional change. In this study, we employed structural covariance networks (SCNs) analysis to investigate abnormalities in the macroscopic topological organization of the brain in individuals with CUD, based on CT measures. A total of 68 patients with CUD and 52 healthy controls (HCs) were included from the OpenNeuro database. The results demonstrated that the CUD group exhibited a significantly reduced global clustering coefficient and a shift in small‐world properties, with network topology becoming more closely aligned with a lattice‐like configuration. In addition, CUD showed disrupted rich‐club connectivity, particularly within core frontoparietal regions. Notably, no significant group differences were observed in network robustness against random failure or targeted attack. Together, these findings indicated an imbalance between network integration and segregation in the structural brain networks of individuals with CUD, providing a novel network‐level perspective on the pathophysiology of addiction and suggesting potential utility as a diagnostic neurobiological marker.

## Graphical abstract

Cocaine use disorder (CUD) disrupts large‐scale brain network coordination. Using structural covariance network analysis, we found reduced local clustering, impaired rich‐club connectivity, and a shift toward lattice‐like organization in CUD patients, while global network robustness remained preserved. These findings reveal a dissociation between local and global network integrity, offering a systems‐level perspective on addiction neurobiology.

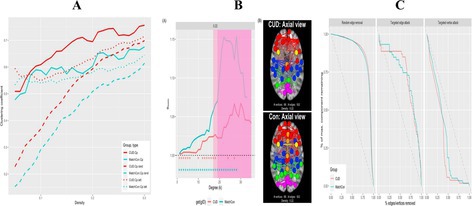

**Abbreviations**
CUD: cocaine use disorder
F: female
HCs: healthy controls
M: male
VBM: voxel‐based morphometry
SBM: surface‐based morphometry
CT: cortical thickness
SCNs: structural covariance networks
CV: cortical volume
SA: surface area
MRI: magnetic resonance imaging
Cp: clustering coefficient
LE: local efficiency
Lp: path length
GE: global efficiency
PC: participation coefficient
WMD: within‐modular degree
CMC: cortical meancurve
FDR: false discovery rate

## 1 Introduction

Cocaine, one of the most widely abused illicit stimulants globally, has precipitated an addiction crisis that now constitutes a severe public health emergency. In 2017, an estimated 18 million people worldwide were reported to have used cocaine [1]. Following a period of regular use, individuals often escalate their consumption, compulsively seeking the drug's reinforcing effects and taking larger doses than before, then progressively culminating in compulsive drug seeking and a loss of control over use [2]. Even among those who undergo treatment, relapse rates remain high, perpetuating the trajectory toward cocaine use disorder (CUD) [3]. The consequences of CUD extend beyond the individual, exacting a toll on both mental and physical health. Affected individuals frequently exhibit impaired emotional regulation [4] and face an elevated risk of severe cardiovascular and cerebrovascular disease [5]. Moreover, CUD is associated with increased aggression [6], which heightens the likelihood of criminal behaviour [7] and imposes broader societal burdens.

Neurobiological research has demonstrated that chronic cocaine exposure leads to persistent neuroadaptive changes within the reward system, motivational circuitry and executive control networks [8], which indicates the whole‐brain pathology of CUD [9, 10]. These adaptations, primarily affecting fronto‐striatal pathways and limbic circuits, are thought to constitute the neural substrate underlying the perpetuation of addictive behaviours and increased relapse risk [11]. Although functional abnormalities in CUD have been extensively investigated, alterations in structural network organization remain poorly understood. Voxel‐based morphometry (VBM) and surface‐based morphometry (SBM) studies have consistently reported grey matter volume reductions or cortical thinning in individuals with CUD, particularly within the prefrontal cortex, orbitofrontal cortex, anterior cingulate cortex, insula and striatum [12, 13]. One study investigating the cortico‐striatal circuitry reported that individuals with cocaine addiction showed decreased functional connectivity between the bilateral caudate nuclei and both the posterior insula and the right postcentral gyrus [3]. Furthermore, patients with CUD exhibited reduced cortical thickness (CT) and cortical volume (CV) in regions including the occipital cortex, fusiform gyrus and insula [13, 14, 15]. Additionally, region‐specific alterations in surface area (SA) were observed, with certain areas showing either increased or decreased SA [14]. Moreno‐López L et al. also found that cocaine‐dependent individuals exhibited volumetric alterations in the striatum and thalamus [15]. Furthermore, structural alterations within fronto‐striatal circuitry are thought to underlie impulsivity and compulsive behaviour, while changes in cortical morphology may reflect neurotoxic effects of chronic substance exposure [16]. However, these investigations have largely relied on regionally confined analyses, which overlook coordinated patterns of morphological variation across distributed brain areas. Given that addiction is increasingly conceptualized as a disorder of interacting large‐scale networks [17], a purely local approach may be insufficient to fully capture its underlying neurobiology.

Structural covariance networks (SCNs) analysis provides a systematic framework for investigating coordinated patterns of brain morphology across regions. CT‐based SCNs typically exhibit small‐world properties and rich‐club organization, reflecting an efficient balance between local specialization and global integration [18]. Aberrant SCNs topology has been documented in various psychiatric and neurological conditions, including Alzheimer's disease [19], schizophrenia [20] and alcohol dependence. In alcohol dependence, researchers have observed reduced network segregation and disrupted hub connectivity, suggesting that chronic substance exposure may remodel the organization of large‐scale structural brain networks [21]. SCNs have been widely used to characterize brain network alterations in neuropsychiatric populations. Beyond substance use disorders, recent studies have applied graph theoretical approaches to structural and functional networks in subthreshold depression [22], revealing convergent alterations in default mode and salience networks. These findings underscore the transdiagnostic potential of network‐based analyses. However, studies examining CT‐based SCNs topology in CUD remain scarce, limiting our understanding of the structural network basis of stimulant addiction.

Accordingly, this study employed graph theoretical approaches to systematically evaluate the topological properties of CT‐based SCNs in individuals with CUD. By characterizing macroscale structural organization from a network perspective, we aim to advance understanding of the neurobiological mechanisms underlying cocaine addiction, utilize graph theory methods to systematically evaluate the global and local topological properties of the SCNs in patients with CUD (such as small‐worldness, rich‐club organization and network robustness) and to identify potential imaging markers for future clinical research. We hypothesized that patients would exhibit reduced network segregation and disrupted hub connectivity relative to healthy controls (HCs) and that the brain networks of patients with CUD will exhibit topological disturbances characterized by impaired local differentiation capacity and damaged connections of core nodes.

## 2 Methods

### 2.1 Participants

In this cross‐sectional study, we used the data from the OpenNEURO database (https://openneuro.org) [23]. Sixty‐eight individuals with CUD were enrolled in this study based on the following inclusion criteria: (1) age between 18 and 50 years old; (2) right‐handed; (3) cocaine dependency with an active consumption of at least twice a week in the last month. The exclusion criteria included the following: (1) current dependence (by DSM‐IV criteria) on other substances (alcohol or nicotine); (2) pregnant or breastfeeding; (3) neurological and psychiatric disorders; (4) with a severe systemic disease such as tumours or digestive system disease; and (5) magnetic resonance imaging (MRI) contraindications. Additionally, 52 matched HCs were enrolled and met the above exclusion criteria. MRI scanning and clinical assessments for all participants were performed once. All participants were asked to respond to an array of questionnaires administered by trained psychologists who were blinded to this study to obtain CUD severity measures and tobacco use. More detailed information about the clinical assessments and informed consent from all participants can be found in a previous study [23, 24].

### 2.2 MRI Data Acquisition

MRI scanning was conducted using a 3.0T MRI scanner (Philips Healthcare, Best, Netherlands & Boston, MA, United States). The head of each participant was held in position using a custom‐built head holder. Structural images were acquired with the following parameters: echo time = 3.5 s, repetition time = 7 s, slice thickness = 1 mm, field of view = 240, matrix size = 240 × 240 mm, voxel size = 1 × 1 × 1 mm, scan time = 3.19 min, and 180 sagittal slices.

### 2.3 MRI Data Analysis

All participants' MRI data were processed with FreeSurfer v7.3.2 (http://surfer.nmr.mgh.harvard.edu), which automatically creates a three‐dimensional model of the cortical surface for morphometric measurements. Briefly, the cortical reconstruction includes motion correction and averaging of multiple volumetric T1‐weighted images, removal of non‐brain tissue with a hybrid watershed/surface deformation procedure, automated Talairach transformation, intensity normalization, tessellation of the grey and white matter boundary, and automated topology correction. Then, individual surfaces were inspected for segmentation errors between grey and white matter, and when needed, manually fixed by a trained technician who was blinded to this study. Surface maps were smoothed with a full‐width half‐maximum Gaussian kernel of 10 mm and aligned across participants using a non‐linear transformation in order to co‐register the cortical folding patterns providing ~160 000 surface vertex points where CT is automatically calculated. The vertices are co‐located across all of the subjects to enable comparisons. Then, the average value of CT within 34 automatically defined cortical parcellations was determined by the Desikan atlas in each hemisphere [25, 26].

### 2.4 SCNs Construction and Graph Theory Metrics

The generation of SCNs and the extraction of graph theory metrics were performed separately for each data set. Prior to the SCNs construction, each scanner‐adjusted ROI was residualized for mean global thickness. For cases and controls independently, SCNs were generated by calculating the Pearson's correlation between every pair of residualized ROIs. The correlation coefficients of each group were binarized according to a series of thresholds following a density‐based approach to ensure SCNs were equal in size (i.e., groups were matched for the number of edges). Thresholds increased in 0.05 increments starting at the minimum density (Dmin), defined as the minimum density at which the networks of both groups remained fully connected, up to a density preserving the top 30% of the edges. Identical density thresholds were applied to both groups during between‐group analyses to ensure comparability of graph theoretical metrics.

Graph theory metrics summarize SCNs organization at several levels of complexity: edge (e.g., correlation coefficients), node (e.g., transitivity) and network (e.g., modularity). Network metrics of segregation and integration were calculated for each group and density. Briefly, greater segregation [i.e., higher clustering coefficient (Cp), local efficiency (LE) or modularity] indicates greater correspondence among adjacent nodes and greater integration [i.e., lower average shortest path length (Lp) or greater global efficiency (GE)] indicates higher correspondence among distant nodes.

We formerly reported a pattern of lower SCNs segregation (lower Cp, LE and modularity) and higher integration (lower Lp and higher GE) in individuals with heavy alcohol use suggesting fewer short‐range edges and more long‐distant edges. To explore whether these phenomena were related, node‐level metrics of transitivity, participation coefficient (PC) and within‐modular degree (WMD) were calculated. Transitivity is a segregation metric reflecting the number of closed triangles per node (i.e., whether neighbours of a node are neighbours of each other). As nodes that are similar in thickness should be assorted to the same module, PC and WMD could clarify why some nodes are poorly segregated. PC captures how similar in thickness anode is to nodes from other modules, where higher values of PC represent more intermodular, long‐distance edges. Conversely, WMD captures how similar in thickness a node is to other nodes in its own module, i.e., the number of intramodular, short‐range edges. Consequently, PC and WMD shed light upon whether a node with low segregation (low transitivity) is caused by more inter modular edges (high PC), fewer intramodular edges (low WMD) or both. All steps were performed in brainGraph version 2.7.3. Rich nodes were defined based on degree levels corresponding to the normalized rich‐club coefficient exceeding 1, indicating the presence of rich‐club organization.

### 2.5 Statistical Analysis

The demographic and clinical characteristics of all participants were analysed using SPSS software, version 25.0. Independent samples *t*‐tests were used to evaluate group differences in age, CUD severity measures and tobacco use, while chi‐square tests were used to measure gender differences.

For anatomical patterns, two sample *t*‐test was conducted to examine the differences in CMC, CT, CV and SA of individuals with CUD in contrast to HCs while controlling for the effects of education level, years of tobacco use and intracranial volume. The significance threshold was set at *p* < 0.05 after false discovery rate (FDR) correction for multiple comparisons across all subcortical structures. The effect size (g) was reported according to Hedges [27], corrected for unequal sample sizes [28]. Effect size is a unit‐free description of the strength of an effect, independent of sample size [29], which could reflect the variance of the structural pattern in this study.

Finally, in order to explore neuroanatomical predictors for CUD status, binary logistic regression analysis was conducted to examine whether abnormal anatomical alterations (brain regions with medium effect size, Cohen's d > 0.5) could predict CUD status. Additionally, 18 linear regression models were performed between the brain anatomical indices and cocaine use features in individuals with CUD. This model controlled the effects of age, sex and other clinical variables as covariates. The significance threshold was set at *p* < 0.05 after FDR correction.

## 3 Results

### 3.1 Demographic and Clinical Characteristics

A total of 68 individuals with CUD and 52 matched HCs were included in the analysis. There were no significant differences between groups in age, sex, or years of education (*p* > 0.05). However, as expected, the CUD group exhibited significantly higher scores on measures of cocaine use severity and tobacco use (*p* < 0.001) (Table 1).

**TABLE 1:** Demographics between groups.

Characteristics	Groups		Statistics	
	CUD (*n* = 68)	HCs (*n* = 52)	t/χ^2^	*p* value
Gender (F/M)	8/60	9/43	χ^2^_1_ = 0.36	0.55
Age (years)	30.56 (7.02)	30.33 (8.06)	t_118_ = 0.17	0.87
Education level (years)	11.32 (3.13)	12.91 (3.45)	t_118_ = 2.62	< 0.05
Onset age of cocaine use	20.66 (5.21)	0.64 (3.21)	t_118_ = 24.42	< 0.01
Years of cocaine use	9.75 (6.46)	0.46 (2.62)	t_118_ = 9.77	< 0.01
Week dose of cocaine use	2.77 (1.22)	0.12 (0.83)	t_118_ = 13.42	< 0.01
Years of tobacco use	12.29 (7.54)	5.64 (7.64)	t_118_ = 4.77	< 0.01

*Note:* Continuous variables are reported as mean (standard deviation).

Abbreviations: CUD, cocaine use disorder; F, female; HCs, healthy controls; M, male.

### 3.2 Global Topological Properties of SCNs

#### 3.2.1 Clustering Coefficient and Small‐World Properties

**FIGURE 1:**
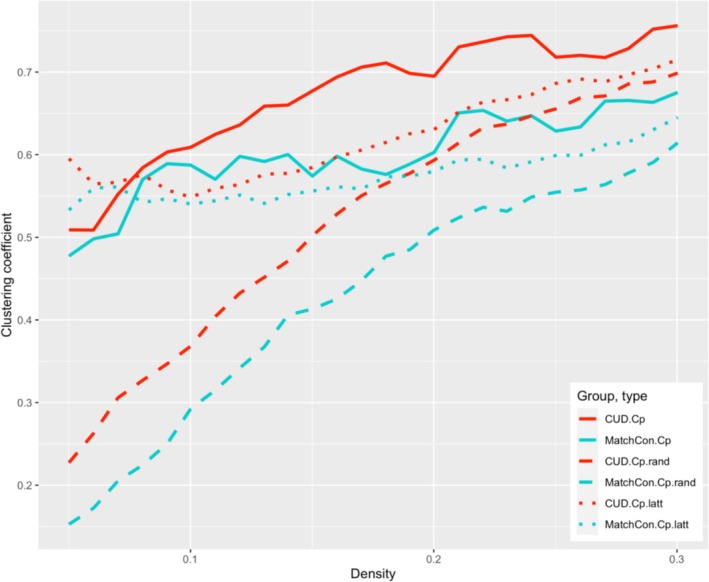
Clustering coefficients for random networks between CUD and HCs. The solid lines are clustering for the observed networks. The dotted line are values from networks generated by the Markov Chain approach, and the dashed lines are values from networks generated by the ‘simple’ approach.

**FIGURE 2:**
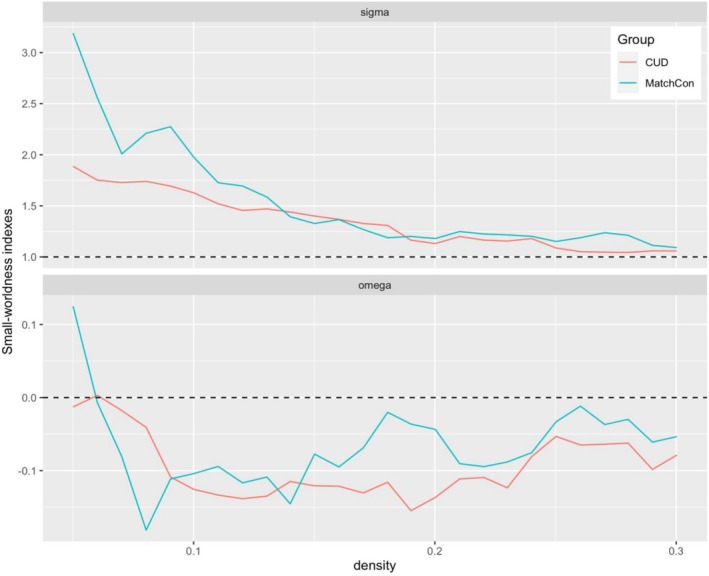
Small‐world indexes. (top) The classic small‐world index, σ; the dashed horizontal line at y = 1 is included to show the minimum value for a network to be considered ‘small‐world’. (bottom) The small‐world index ω; the dashed horizontal line at y = 0 indicates the value at which the network displays a balance between clustering coefficient and characteristic path length. Networks with ω closer to 0 indicate more balance between high clustering and low path length; networks with ω closer to −1 are more similar to a lattice network.

Compared with HCs, the CUD group showed significantly lower clustering coefficients across a range of network densities, indicating reduced local network segregation (see Figure 1). Small‐world analysis further revealed that CUD networks were structurally closer to lattice‐like organization at higher densities, as evidenced by lower values of the classical small‐world index σ and a shift of the balance index ω toward −1 (see Figure 2).

#### 3.2.2 Rich‐Club Organization

**FIGURE 3:**
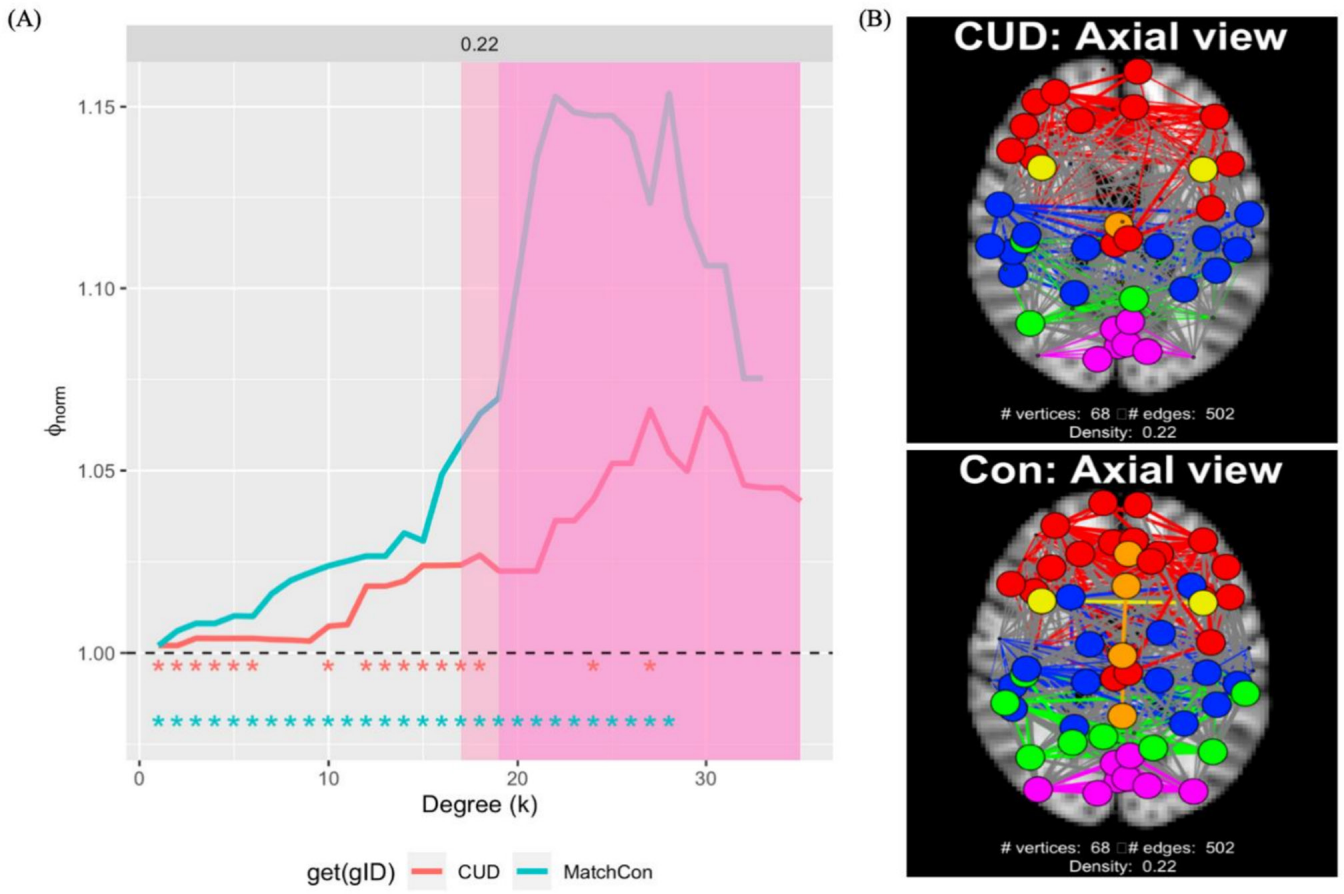
(A) normalized rich club coefficient vs. degree and (B) rich‐club regions for CUD and Con at a density of 0.22.

At a network density of 0.22, the normalized rich‐club coefficient was significantly lower in the CUD group compared with HCs (*p* < 0.05). Compared with HCs, CUD exhibited lower normalized rich‐club coefficients at a density of 0.5, indicating weakened connectivity among highly connected hub regions. Spatial mapping of rich‐club regions demonstrated widespread involvement of frontal and parietal hubs in CUD, suggesting impaired connectivity among highly connected core regions (see Figure 3).

#### 3.2.3 Network Robustness to Attacks

**FIGURE 4:**
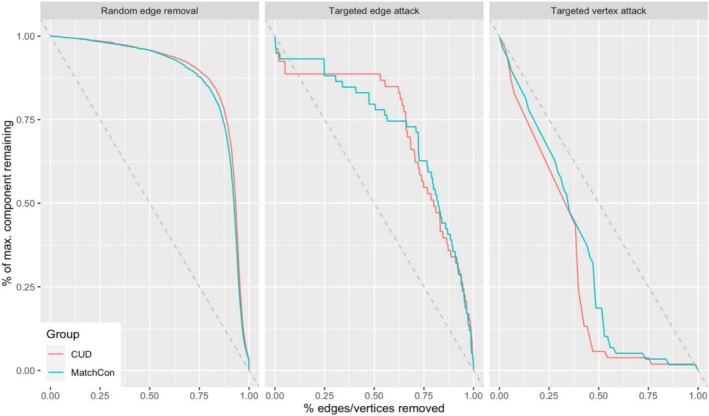
Robustness analyses for the CUD and HCs groups at a density of 0.22.

Despite alterations in local and hub‐level topology, network robustness analyses showed no significant differences between CUD and HCs. Both groups exhibited similar resilience to random failure of edges or vertices, as well as to targeted attacks on high‐degree nodes. The decline of the largest connected component followed comparable trajectories across attack scenarios in both groups (see Figure 4).

### 3.3 Regional Anatomical Alterations

After controlling for education, tobacco use, and intracranial volume, individuals with CUD showed reduced CT in several regions, including the prefrontal cortex, anterior cingulate and insula (FDR‐corrected *p* < 0.05, effect size *g* > 0.5). Binary logistic regression indicated that these structural abnormalities were significant predictors of CUD status (model *p* < 0.01).

#### 3.3.1 Clustering Coefficients for Random Networks

The random graphs generated by the Markov Chain process (dotted lines) have a similar clustering level as the observed networks (solid lines). As expected, the ‘simple’ random graphs have much lower clustering. Compared with HCs, CUD showed lower clustering coefficients for random networks (Figure 1).

#### 3.3.2 Small‐World Index

The results of this small‐world analysis indicated that the CUD group's networks at higher density were closer to a lattice than the HCs' networks (Figure 2).

#### 3.3.3 Rich Club Index

Compared with HCs, CUD exhibited lower normalized rich club coefficient at a density of 0.22 (Figure 3A). Moreover, CUD showed widespread rich‐club regions, including frontal and parietal regions (Figure 3B).

#### 3.3.4 Network Robustness

Both groups' networks were resilient against random failure of vertices and edges, and were similarly resilient to targeted attacks of vertices and edges (Figure 4).

## 4 Discussion

To our knowledge, this study is the first to integrate SBM with graph theoretical approaches to systematically reveal aberrant topological patterns of CT‐based SCNs in individuals with CUD [30, 31]. Our core finding is that individuals with CUD exhibit a reduced clustering coefficient in their brain networks along with a shift in small‐world properties toward a more lattice‐like configuration. This phenomenon reflects a disruption in the efficient balance between local segregation and global integration of information within the brain [18, 32, 33]. These findings not only supplement existing knowledge of the neuroanatomical basis of CUD but also provide a novel perspective for understanding the network‐level pathophysiological mechanisms underlying stimulant addiction [8, 9, 16]. It should be noted that SCNs derived from CT reflect coordinated interregional morphological variation across individuals rather than direct anatomical or white matter connectivity. This aligns with findings from previous structural neuroimaging studies, which have largely reported focal grey matter atrophy and cortical thinning in individuals with CUD [13, 15, 34, 35, 36]. Chronic cocaine exposure induces neuroadaptive changes, including aberrant synaptic pruning and neuronal loss [2, 11, 37], which may directly contribute to weakened connectivity between local brain regions, consequently diminishing the efficiency of local information processing. From a clinical standpoint, this local network dysfunction may help explain the functional deficits patients with CUD display in fine sensory discrimination, sustained attention and the execution of specific local tasks, as these cognitive functions rely on the efficient coordination of local neural circuits. For instance, the prefrontal cortex, a critical region for executive function, exhibits not only reduced CT but also decreased local clustering in patients with CUD, potentially constituting a key neural substrate for their difficulty in inhibiting impulsive drug‐seeking behaviour [8, 13, 15]. Notably, the clinical relevance of nodal clustering coefficient extends beyond substance use disorders. A very recent study by Suo et al. [38] demonstrated that individual differences in intolerance of uncertainty—a transdiagnostic risk factor for psychological distress—were positively associated with nodal clustering coefficient patterns within limbic and default mode networks in healthy young adults. Taken together with our finding of decreased clustering in CUD, these contrasting directions suggest that the relationship between clustering coefficient and behavioural phenotypes may be disorder‐ or trait‐specific: heightened modular connectivity in certain networks may reflect adaptive or vulnerability processes for anxiety‐related traits, whereas reduced nodal clustering in CUD may index neurotoxic effects of chronic cocaine exposure on local information processing capacity. The disruption of rich‐club organization, particularly the aberrant connectivity of key nodes within the frontoparietal control network, provides a structural basis for the impaired ‘top–down’ cognitive control characteristic of CUD. Frontoparietal regions, including the dorsolateral prefrontal cortex and the inferior parietal lobule, serve as core nodes of the rich‐club network and are crucial for higher‐order cognitive functions such as decision‐making, impulse control and goal‐directed behaviour [17, 20, 32]. Our results demonstrate a significantly reduced normalized rich‐club coefficient in patients with CUD, indicating weakened connectivity strength among these highly connected hub regions. This finding converges with previous functional neuroimaging studies reporting hypoactivation and disrupted functional connectivity within the frontoparietal network during cognitive control tasks in CUD [3, 4, 39]. Impaired rich‐club connectivity may disrupt the coordinated interaction between cognitive control and reward systems, allowing the motivational drive for drug reward to override the inhibitory control exerted by the prefrontal cortex, ultimately leading to compulsive drug use and high relapse rates [8, 16, 37].

Despite significant topological alterations at local and hub levels, network robustness in patients with CUD did not significantly differ from that of HCs. Both groups exhibited similar resilience when facing random failure of edges or nodes and targeted attacks on high‐degree nodes. This finding may reflect relative preservation of global network resilience and may suggest potential compensatory network organization. Even when specific circuits are compromised by cocaine exposure, the remaining network structure may still maintain basic functional integrity, averting systemic collapse. This robustness may stem from the brain's structural plasticity, such as compensatory reinforcement of alternative pathways or the preservation of low‐degree connections not directly involved in addiction‐related networks. However, it is important to clarify that this robustness is relative; while it may prevent catastrophic network failure, it does not compensate for the specific cognitive and behavioural deficits resulting from damage to core hubs like those in the frontoparietal network.

Furthermore, our study found that reduced CT in regions such as the prefrontal cortex, anterior cingulate cortex and insula were significant predictors of CUD status, and that dorsolateral prefrontal cortex thickness was negatively correlated with years of cocaine use. This aligns with previous structural neuroimaging studies reporting cortical thinning and grey matter abnormalities in cocaine‐dependent individuals [13, 15, 34, 35, 36, 40, 41]. This aligns with our previous research suggesting that aberrant neuroanatomical patterns in CUD hold potential diagnostic value [14, 24]. The negative correlation between CT and duration of cocaine use implies a progressive nature of cocaine‐induced structural damage: prolonged drug exposure exacerbates brain structural abnormalities, which in turn may reinforce addictive behaviours, creating a vicious cycle [42, 43]. This dose‐dependent effect underscores the importance of early intervention in patients with CUD; timely cessation of cocaine use might mitigate further structural damage and improve treatment outcomes.

Comparing our findings with those from studies on other substance use disorders reveals intriguing commonalities and differences. Ottino‐González et al. also reported reduced SCN segregation and disrupted rich‐club connectivity in alcohol dependence [21], suggesting that different substance addictions may share common network‐level abnormalities. This might reflect a universal ‘addiction phenotype’, characterized by impaired cognitive control and disrupted brain network balance [8, 9]. However, differences exist: patients with alcohol dependence often exhibit more widespread cortical thinning in temporal and occipital lobes, whereas structural alterations in CUD appear more concentrated in frontoparietal‐related networks [21, 36]. These disparities may be related to the distinct neuropharmacological actions of cocaine and alcohol. Cocaine primarily acts on dopamine transporters, leading to dopamine overflow in the striatum and frontoparietal regions [11, 37], while alcohol exerts broader effects on GABAergic and glutamatergic systems across multiple brain regions. Such cross‐disorder comparative studies are valuable for identifying both addiction‐specific and transdiagnostic neuroanatomical markers, informing the development of targeted intervention strategies.

This study has several limitations that warrant consideration in future research. First, the cross‐sectional design precludes establishing causality between brain network abnormalities and CUD. It remains unclear whether the observed SCNs alterations represent pre‐existing vulnerability factors for cocaine addiction or are consequences of chronic cocaine exposure [35, 36]. Longitudinal studies tracking high‐risk individuals (e.g., those with a family history of addiction) before and after cocaine initiation are needed to clarify this issue. Second, although we controlled for potential confounders like tobacco use and education level, other unmeasured variables such as comorbid psychiatric disorders, nutritional status and socioeconomic factors may have influenced the results. Given the significant between‐group differences in tobacco use, the potential confounding effects of smoking on CT and structural covariance organization should be carefully considered when interpreting the present findings. Future studies with larger samples and better‐matched control groups (e.g., non‐smoking participants with CUD or smoking‐matched controls) are needed to disentangle the specific effects of cocaine from those of nicotine. Moreover, although the sample size was comparable with several previous SCN studies in neuropsychiatric disorders, graph theoretical analyses may still be sensitive to sample size, and therefore the present findings should be interpreted with appropriate caution. Expanding the sample size and incorporating multi‐centre data would enhance the generalizability of our findings. Meanwhile, we acknowledge that correlational analyses between global network metrics (e.g., clustering coefficient, rich‐club coefficient) and CUD severity measures (e.g., years of cocaine use, weekly dose, onset age) were not performed in the current study. Our primary aim was to characterize group‐level topological differences between CUD and HCs. Future studies with larger sample sizes should examine these associations to determine whether the observed network‐level alterations track with clinical severity, which would further support the potential utility of graph theory metrics as prognostic or monitoring biomarkers in CUD. Last, our analysis focused exclusively on CT‐based SCNs. Future studies should integrate multiple structural indices, such as white matter fibre tract integrity and functional connectivity data, to gain a more comprehensive understanding of brain network reorganization mechanisms in CUD.

## 5 Conclusions

In summary, this study demonstrated that CUD was associated with pronounced topological abnormalities in SCNs, characterized by reduced local segregation, disrupted rich‐club connectivity and altered small‐world architecture. These network‐level alterations are closely linked to the clinical phenotype of CUD and may hold promise as neurobiological markers to aid in diagnosis and monitoring of treatment response.

## Funding

This work was supported by the Natural Science Foundation of Zhejiang Province (LTGY23H180011), the Medical Health Science and Technology Project of Zhejiang Provincial Health Commission (No. 2023KY91), and the Wenzhou Municipal Fundamental Research Project (No. Y2023535).

## Conflicts of Interest

The authors declare no conflicts of interest.

## Data Availability

The data that support the findings of this study are available from the corresponding author upon reasonable request.
